# Actual Anatomical and Dosimetric Changes of Parotid Glands in Nasopharyngeal Carcinoma Patients during Intensity Modulated Radiation Therapy

**DOI:** 10.1155/2015/670327

**Published:** 2015-02-22

**Authors:** Gang Ren, Shou-Ping Xu, Lei Du, Lin-Chun Feng, Bao-Lin Qu, Hai-Xia Liu, Chuan-Bin Xie, Lin Ma

**Affiliations:** ^1^Department of Radiation Oncology, Chinese PLA Air Force General Hospital, 30 Fucheng Road, Beijing 100142, China; ^2^Department of Radiation Oncology, Chinese PLA General Hospital, 28 Fuxing Road, Beijing 100853, China; ^3^Department of Radiation Oncology, Hainan Branch of Chinese PLA General Hospital, Haitang Bay, Sanya 572000, China

## Abstract

The goal of this study was to evaluate the actual anatomical and dosimetric changes of parotid glands in nasopharyngeal carcinoma patients during intensity modulated radiation therapy. With helical tomotherapy, its planning system, and adaptive software, weekly anatomical and dosimetric changes of parotid glands in 35 NPC patients were evaluated. Interweekly parotid volume varied significantly (*P* < 0.03). The rate of volume change reached the highest level at the 16th fraction. The average *V*_1_ increased by 32.2 (left) and 28.6 (right), and the average *D*_50_ increased by 33.9 (left) and 24.93 (right), respectively. Repeat data comparison indicated that the *V*_1_ and *D*_50_ varied significantly among different fractions (both with *P* = 0.000). The variation of parotid volume was inversely correlated with that of the *V*_1_ and *D*_50_ (both with *P* = 0.000). In conclusion, parotid volume and actual dose vary significantly in NPC patients during IMRT. Replanning at the end of the fourth week of IMRT may have clinical benefits.

## 1. Introduction

Due to the anatomical and biological specificity of nasopharyngeal carcinoma (NPC), radiation therapy or chemoradiotherapy has been recognized as a definitive treatment [1]. Studies have shown that the higher the radiation dose delivered to the target volume, the better the local disease control ratio [2]. The escalation of the delivered dose, however, often leads to severe and related side effects. Xerostomia is one of the most frequent side effects and the amount of radiation that is delivered to the parotid glands, which assume a major role in stimulating salivary flow, affects NPC patients' quality of life. Therefore, it is crucial to minimize the dose to the parotid gland while assuring adequate dose distribution to the target volume in the treatment of NPC. Unlike two-dimensional conventional radiation therapy (2DCRT) and three-dimensional conformal radiation therapy (3DCRT), intensity modulated radiation therapy (IMRT) can deliver a highly conformal dose to targets while effectively sparing critical normal organs, potentially improving the local control rate and reducing radiation-related toxicities [3, 4].

Patients with head and neck cancer may be subjected to significant anatomical changes during radiation therapy, changes which can cause volume shrinkage near the facial surface. And parotid gland variations may result in an unanticipated overdose. A hybrid IMRT plan, generated by applying the beam configurations of the first plan to the anatomical structures of the second simulation CT images, has been used to evaluate possible volumetric and dosimetric variations [5, 6]. However, it is inevitable that this approach will develop bias.

Helical tomotherapy (HT) is a unique IMRT modality that combines elements of diagnostic radiology and radiation therapy in a single unit. In addition to its ability to deliver a highly conformal dose distribution, HT is equipped with xenon detectors that have been designed to obtain the megavoltage computed tomography (MVCT) images that are used for pretreatment setup verification [7]. Meanwhile, HT is equipped with adaptive planning software which can calculate actual dose distribution in each treatment fraction [8]. To evaluate the actual anatomical and dosimetric changes in NPC patients during IMRT, we performed this study.

## 2. Materials and Methods

Between March 2009 and August 2010, 35 histologically proven and locoregionally advanced NPC patients were treated with HT in our center. Informed consent was obtained from all patients before receiving treatment. Patient characteristics are summarized in Table 1. All patients were treated with HT, but 15 underwent concurrent cisplatin-based chemotherapy, 2 received concurrent cisplatin-based chemotherapy with anti-EGFR monoclonal antibody (Mab), and 5 underwent concurrent anti-EGFR Mab treatment. Patients' weight was noted before and at the end of treatment.

All patients underwent planning kilovoltage CT (KVCT) scanning with a slice thickness of 3 mm. The patients were immobilized with a thermoplastic head-and-shoulder mask and a head-and-shoulder immobilization board. Each patient underwent scanning through the head and neck region (from the head to below the clavicles). Enhancement CT and plain CT images were transmitted to a Pinnacle^3^ 8.0 workstation and fused. Enhanced CT, MRI, or PET-CT images were used to guide the contours of the target volumes. Each patient received a total of 33 fractions of radiation, resulting in 70 Gy to the gross tumor volume and positive lymph nodes (pGTVnx and pGTVnd were obtained by expanding the corresponding gross tumor volume and metastatic nodes with a margin of 3–5 mm), 60 Gy to the high-risk planning target volume, and 50–56 Gy to the low-risk planning target volume. Treatment planning was made on a TomoTherapy Hi-Art 2.2.4.1 workstation. The physician and physicist simultaneously decided whether treatment planning would be executed. No more than 5% of PTV volume received more than 110% of the prescribed dose. Dose-volume constraints for OARs were utilized similarly to the previous published paper [9]. The primary dosimetric parameters of main target volumes and organs of risk are shown in Table 2.

During HT therapy, patients underwent MVCT guidance at least once every week. To minimize unnecessary irradiation and to reduce in-room time, the range of MVCT scans included the entire length of parotid glands and the gross tumor (slice thickness was 6 mm). The requisite time depended on the selected range and pitch and was generally about 3 minutes. The patient setup verifications were completed through the automatic and manual coregistration of the on-set MVCT images with the planning CT images based on bony and tissue anatomy.

HT's adaptive software calculated the volume and actual dose distribution according to the pretreatment MVCT scanning. The MVCT images of the first fraction were collected, followed by additional 7 fractions (fractions numbers 6, 11, 16, 21, 26, 31, and 33) for a total of 8 series of images. According to previously noted setup errors, each patient's MVCT images were merged with each patient's corresponding KVCT images using the adaptive software. The same physician manually contoured the parotid glands of each patient on the MVCT images. According to the contoured images, the actual single-fraction dose-volume histograms of the parotid gland were gained in the adaptive software. The volume and dosimetric parameters were recorded on the basis of the dose-volume histograms.

The volumes of the left and right parotids were calculated 8 times and the ratios to their volumes before the first fraction were calculated for comparison. The inside and outside target volumes were also obtained. The actual doses of each single fraction including the *V*_1_ (the relative volume of the parotid gland that received 1 Gy) and *D*_50_ (half parotid gland receiving dose) were recorded. The distances between the outside borders of the bilateral parotid glands and the facial transverse diameter at the level of the odontoid process and the root of the C2 vertebral body were measured.

Spearman's correlation analysis was used to study the correlation between the two series of parameters. The parotid volume before the first fraction of radiation therapy, as measured using MVCT images, and that from the primary KVCT planning images were compared using the paired Wilcoxon rank sum test. The interfractional parameters affecting these variations were studied using repeated measures and linear regression analyses.

## 3. Results

### 3.1. Variations of Parotid Volume

**Figure 1:**
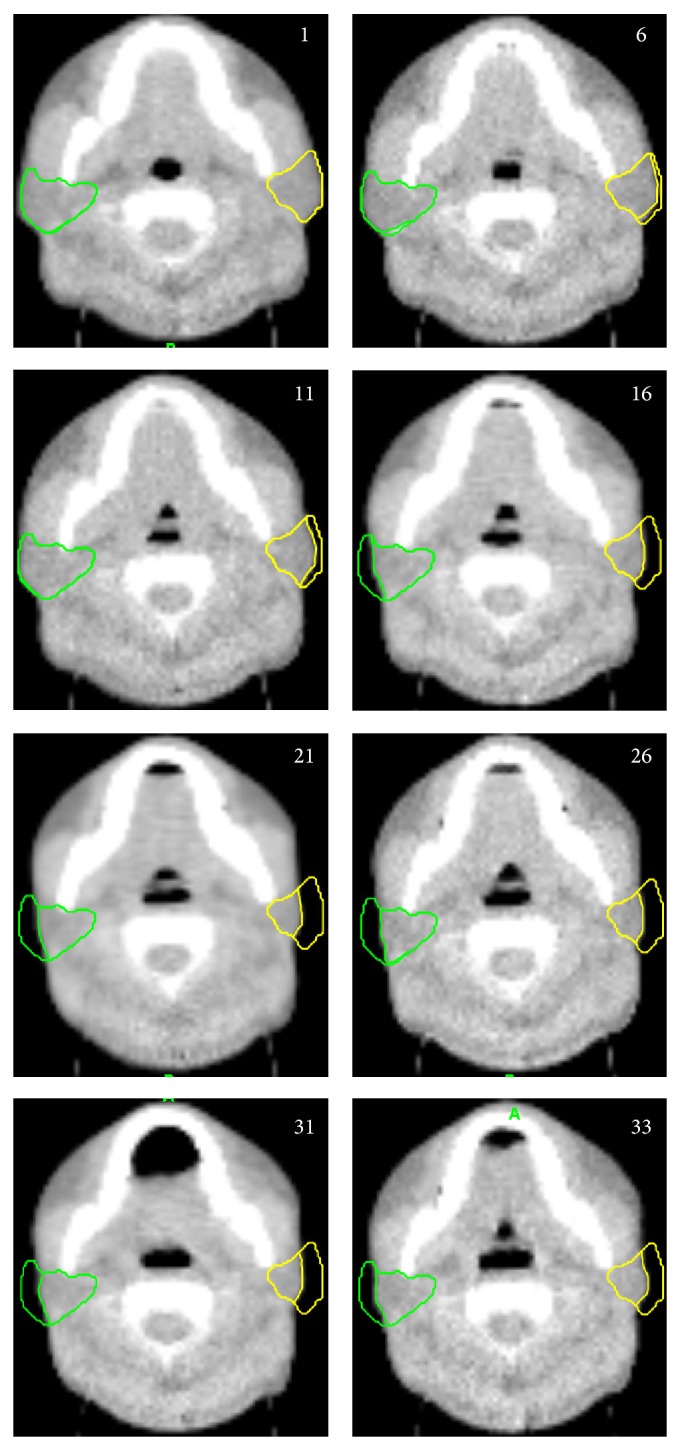
MVCT images of one patient showing parotid volume variations during radiation therapy (the number on each image is the fraction number).

Each patient had 8 series of MVCT fusion images, and a total of 280 series of images were gathered for the 35 patients. There was no significant difference in parotid volume between the MVCT images before the first fraction and the KVCT images of the initial plans (*Z* = −0.961, *P* = 0.337). The parotid volume gradually decreased during radiation therapy (Figure 1). Before the first fraction, the volumes of the left and right parotid glands were 29.43 ± 11.6 cm^3^ (12.98–65.19 cm^3^) and 29.03 ± 10.55 cm^3^ (12.80–53.11 cm^3^), respectively. Before the last fraction, the volumes of the left and right parotid glands were 21.02 ± 11.07 cm^3^ (8.70–63.77 cm^3^) and 22.28 ± 9.67 cm^3^ (7.08–51.87 cm^3^), respectively. When measured as a percentage of the initial volume at the end of radiation therapy, the average volume reduction was 29.47% and 24.47% for the left and right parotid glands, respectively. Repeat data comparison indicated that parotid volumes varied significantly every week (*P* < 0.03, Table 3). The rate of volume variation changed during radiation therapy, reaching its peak at the 16th fraction and later decreasing. The left and right parotid volumes had an average reduction of 0.26 cm^3^ (0.92%)/treatment day and 0.22 cm^3^ (0.76%)/treatment day, respectively. At the end of radiation therapy, the patients' weight lost 11.5 ± 5.75% (−2.94–27.59%). Body weight changes correlated with that of parotid volume (*r* = 0.418, *P* = 0.012). At the last fraction of treatment, patients' facial diameter was decreased by 9.49 ± 3.94% (1.66–17.1%), without correlation with parotid volume changes (*r* = 0.236, *P* = 0.172).

### 3.2. Displacement of Parotid Glands

The left and right parotid glands shifted medially during radiation therapy. The distance between the bilateral parotid external borders was 14.60 ± 1.14 cm (12.28–17.24 cm) and 13.52 ± 1.31 cm (10.93–17.24 cm) before the first and last fractions, respectively; the average variation was −7.5 ± 3.85%.

The average ratio of the intratarget volume to the extratarget volume of the left parotid gland increased from 0.28 ± 0.19 (0.03–0.87) to 0.53 ± 0.42 (0.07–2.2). The average ratio of the intratarget volume to the extratarget volume of the right parotid gland increased from 0.26 ± 0.16 (0.01–0.63) to 0.44 ± 0.34 (0.05–1.81). The average ratio of the intratarget volume to the extratarget volume of the left and right parotid glands increased by 102.3 ± 80.05% and 86.43 ± 122.1%, respectively.

### 3.3. Variations of Parotid *V*_1_

The *V*_1_ of both parotid glands increased gradually during treatment process. The *V*_1_ of the left parotid gland was 38.19 ± 10.56% (21.26–64.08%) and 49.21 ± 12.48% (24.36–80.47%) before the first and final fractions, respectively. The *V*_1_ of the right parotid gland was 35.46 ± 9.37% (11.44–55.52%) and 44.5 ± 12.08% (23.03–69.26%) before the first and final fraction, respectively. When measured as a percentage of the initial volume at the end of treatment, the average *V*_1_ increased by 32.2% and 28.6% in the left and right parotid glands, respectively. The volume had an average increase of 0.35% (1.0%)/treatment day and 0.28% (1.06%)/treatment day for the left and the right parotid glands, respectively. Repeat data comparisons indicated that the *V*_1_ varied significantly among different fractions (*P* = 0.000).

### 3.4. Variations of Parotid *D*_50_

The *D*_50_ of both parotid glands increased gradually during treatment process. The *D*_50_ of the left parotid gland was 0.791 ± 0.253 Gy (0.54–1.55 Gy) and 1.04 ± 0.348 Gy (0.607–2.0 Gy) before the first and final fractions, respectively. The *D*_50_ of the right parotid gland was 0.733 ± 0.143 Gy (0.509–1.13 Gy) and 0.928 ± 0.331 Gy (0.569–2.02 Gy) before the first and final fractions, respectively. When measured as a percentage of the initial volume at the end of treatment, the average *D*_50_ increased by 33.9% and 24.93% in the left and right parotid glands, respectively. The volume had an average increase of 0.77 cGy (1.0%)/treatment day and 0.6 cGy (0.78%)/treatment day for the left and right parotid glands, respectively. Repeat data comparison indicated that the *D*_50_ varied significantly among different fractions (*P* = 0.000).

### 3.5. Correlation between Parotid Volume and Dose

During radiation therapy, there was a negative correlation between the parotid volume and the *V*_1_ (*r* = −0.982, *P* = 0.000) and between the parotid volume and the *D*_50_ (*r* = −0.987, *P* = 0.000).

## 4. Discussion

As the most important large salivary glands, parotid glands secrete 60–65% of total saliva volume. After exposure to a high dose of irradiation, the secretary function of the parotid gland is impaired and saliva secretion decreases. Xerostomia thus becomes the main complication in head and neck cancer patients who have received radiation therapy [10]. IMRT represents a new generation of technology and, as compared with 2DCRT and 3DCRT, has better dosimetric advantages, improved conformity and uniformity of treatment targets, and better-protected OARs [4]. Phase III clinical trials showed that IMRT reduces the incidence of xerostomia and improves the quality of life for patients with head and neck cancer [11], but severe dry mouth symptoms still sometimes occur [3]. Xerostomia can be caused by the actual parotid dose increases that result from changes in organ anatomy, tumor size, and body weight that take place during radiation therapy, even when image-guided techniques are used. Not only can these changes cause target underdose, but also overdose to OARs can result in additional complications. To compensate for these changes, replanning can be performed during radiation therapy. Compared to the volume changes in other salivary glands, those in the parotid glands are critical, and studying the pattern of their volume changes may be helpful to decide the replanning timing.

At the end of fractionated radiation therapy, parotid volume decreases, with an average volume reduction of 21.3–42% and an average reduction rate of 0.4–1.4%/day [12–14]. However, some studies showed that parotid volumes had small changes during the first 3-4 weeks [14, 15] and stabilized after the 5th week [3]. Wang et al. [13] reported on a group of head and neck cancer (mainly consisting of oral cavity cancer) patients with postoperative radiation therapy and found that their parotid volume changes were more evident in the first 3 weeks than in the last 3 weeks. The average reduction was 20.01% and 8.57%, respectively, and the average parotid volume had no significant changes 2 and 6 months after radiation therapy as compared with those at the end of treatment. Our study found that parotid volume variation presented a linear pattern throughout radiation therapy, and the rate of volume variation reached its peak at the 16th fraction and then decreased gradually. This can be explained by the use of HT technology and different treatment protocols which combined radiation therapy with chemotherapy or anti-EGFR Mab in locoregional advanced diseases.

In addition to volume reduction, parotid glands also move to the body midline during radiation therapy. Wang et al. [16] studied the parotid displacement in 15 NPC patients at the 18th fraction and found that the center of the left and right parotid glands moved to the body midline with a median motion distance of 4.8 mm and 4.3 mm, respectively. The distance between the outside boundaries of the bilateral parotid glands varied significantly at the end of radiation therapy (*P* < 0.001), with an average reduction of 9.2 mm (0.4–15.2 mm), while the inner boundary distance did not reach significance (*P* = 0.555). Robar et al. [17] studied the parotid anatomical changes every week during radiation therapy in 15 head and neck cancer patients and found that despite the movement of the outside boundaries to the midline (with an average of 2.6 mm and 1.9 mm for the left and right parotid glands, resp.), the parotid centers remained unchanged. Vásquez Osorio et al. [18] measured the parotid displacement in three-dimensional directions in 10 oropharyngeal cancer patients treated with a nonrigid registration technique. CT scanning was executed in the 23rd fraction and they found that changes in the central region of parotid glands were minimal, while changes in the peripheral region were the largest, with an average value of 1 ± 3 mm and 3 ± 3 mm, respectively. In this study, at the end of radiation therapy, the external diameters of the parotid glands obviously shrunk, and the ratios of the intratarget volume to the extratarget volume of both parotid glands increased, results which were similar to those of the previously mentioned studies that indicate that volume reduction was the main cause of parotid displacement.

During radiation therapy, parotid glands, the tumor, and surrounding tissue shrink, deform, and shift to the body midline, leading to variations of the actual parotid dose [12, 19]. Robar et al. [17] sketched parotid glands using weekly KVCT scanning. The initial planning parameters were transplanted to new KVCT images to form new plans. They found that the mean dose (*D*_mean_) of the left and right parotid glands increased by 2.6 ± 4.3% and 0.2 ± 4.0% and *V*_26_ increased by 3.5% ± 5.2% and 0.3% ± 4.7%, respectively, as compared with the initial plan. In this study, the left and right parotid glands received different actual doses, suggesting that multiple factors affected parotid dose variations, such as beam distribution and tumor and metastatic lymph node locations. Using HT with MVCT scanning and adaptive software, Han et al. [12] detected a single actual dose of the parotid gland in 5 NPC patients and found that parotid *D*_50_ was 83.0 ± 28.3 (53.6–151.1) cGy in the first fraction and increased to 142.6 ± 47.3 (72.2–207.9) cGy in the last treatment, which was equivalent to 177 ± 49% of that of the initial plan (97–249%, *P* = 0.0005) and an average increase of 1.7 cGy/fraction. With similar methods, You et al. [20] assessed the single dose in the last week of radiation therapy in 31 head and neck cancer patients and showed that the relative volume receiving 0.75 Gy was increased by 23.6%.

We assessed the actual parotid *V*_1_ and *D*_50_ with weekly MVCT scanning. And the dose variation was less than that reported by Han et al., a difference that was probably due to this study's large number of patients, heterogeneous tumor staging, and different treatment protocols. Replanning during radiation therapy can reduce the dose to OARs and improve the dose distribution to the tumor volume. Wang et al. [5] practiced replanning before the 25th fraction in 28 NPC patients and found that, compared with the initial plan, CTV dose increased by 4.91 ± 10.89% (*P* = 0.024), while the *D*_max⁡_ of the spinal cord and *D*_mean_ and *V*_30_ of the parotid gland decreased by 5.00 ± 9.23 Gy (*P* = 0.008), 4.23 ± 10.03 Gy (*P* = 0.034), and 11.47% ± 18.89% (*P* = 0.003), respectively. Yan et al. [21] recommended replanning after 20 fractions of treatment.

In summary, during the IMRT of NPC, some patients' parotid volumes and locations varied significantly, generally causing an increase of the actual delivered dose. It is thus necessary to identify relevant factors that affect these changes. Our study suggests that replanning is appropriate in the fourth week of IMRT.

## Figures and Tables

**Table 1:** Patients' clinical characteristics.

Parameter	Number	%
Gender		
Male	28	80%
Female	7	20%
Age	11–80 y (median 44 y)	
Tumor stage		
I-II	17	48.6%
III-IV	18	51.4%
Cervical lymph node metastasis		
No	8	22.8%
Yes	27	77.2%

**Table 2:** Planning dosimetric parameters of targets and organs at risk.

Parameter	Average	Standard deviation
PTV1 *D*_95_ (Gy)	60.56	0.71
PTV2 *D*_95_ (Gy)	56.12	1.35
pGTVnx *D*_95_ (Gy)	70.76	1.09
pGTVnd *D*_95_ (Gy)	70.76	0.671
Left parotid		
Volume (cm^3^)	29.67	11.55
*D*_mean_ (Gy)	31.28	4.63
*V*_30_ (%)	40.47	8.83
Right parotid		
Volume (cm^3^)	30.33	6.54
*D*_mean_ (Gy)	30.72	4.39
*V*_30_ (%)	38.87	8.95
Brainstem *D*_max⁡_ (Gy)	53.36	8.76
Left lens *D*_max⁡_	3.94	1.11
Right lens *D*_max⁡_	4.17	1.34
Left optic nerve *D*_max⁡_	33.65	17.48
Right optic nerve *D*_max⁡_	36.1	19.61
Left temporomandibular joint *D*_max⁡_	57.28	7.96
Right temporomandibular joint *D*_max⁡_	56.35	11.56
Left inner ear *D*_max⁡_	63.99	5.05
Right inner ear *D*_max⁡_	63.83	4.76
Oral cavity *V*_40_	32.49	7.31
Larynx-esophagus-trachea *V*_40_	35.02	12.93

*D*_95_: dose delivered to 95% of the target volume; *D*_mean_: mean dose; *D*_max⁡_: maximum dose; *V*_30_: the relative volume of the organ receiving 30 Gy; *V*_40_: the relative volume of the organ receiving 40 Gy.

**Table 3:** Variations of parotid volumes among different fractions (pairwise comparisons).

Fraction number		Average volume variation (cm^3^)	*P*
**1**	**6**	−0.84	0.023
**6**	**11**	−1.3	0.000
**11**	**16**	−1.86	0.000
**16**	**21**	−1.37	0.000
**21**	**26**	−0.99	0.002
**26**	**31**	−0.75	0.02
**31**	**33**	−0.58	0.447
